# Knowledge and attitudes towards psoriasis patients among adults: a cross-sectional study from Turkiye^☆^

**DOI:** 10.1016/j.abd.2025.501229

**Published:** 2025-11-07

**Authors:** Erman Kavlu, Esra Ağaoğlu, Bengisu Karagöz, Hilal Kaya Erdoğan, Muhammed Fatih Önsüz, Selma Metintaş

**Affiliations:** a Department of Public Health, Faculty of Medicine, Eskişehir Osmangazi University, Eskisehir, Turkey; b Department of Dermatology and Venereology, Faculty of Medicine, Eskişehir Osmangazi University, Eskisehir, Turkey

**Keywords:** Attitudes, Health literacy, Knowledge, Psoriasis, Public awareness, Stigmatization

## Abstract

**Background:**

Misconceptions about psoriasis can negatively influence attitudes toward people diagnosed with the condition as in other developing countries. Health literacy is known to be low, which may further exacerbate negative attitudes toward psoriasis patients.

**Objectives:**

The study aimed to evaluate the attitudes and knowledge levels and associated factors regarding psoriasis among adults in a community-based sample.

**Methods:**

This cross-sectional study was conducted among 715 individuals (aged ≥ 18 years) who presented to a university hospital.

**Results:**

Approximately 60% of the participants in the study did not have sufficient knowledge about psoriasis, and approximately half of them had a negative attitude according to the attitude scale. A moderate negative correlation was found between the psoriasis knowledge score and the psoriasis attitude scale score. According to the multivariate linear regression model, predictors of a positive attitude toward psoriasis were identified as having an income-generating job (Beta; 95% CI: -1.812; -3.052 to -0.572), having heard of psoriasis in medical terminology (-3.946; -5.374 to -2.518), being aware of psoriasis (-3.961; -5.518 to -2.404), having a family member or close individual with a psoriasis diagnosis (-3.961; -4.637 to -1.694), and having an adequate knowledge level regarding psoriasis (-2.880; -4.072 to -1.687) (*F* = 22.921, p ≤ 0.001, *R*^2^ = 0.206).

**Study limitations:**

Due to its cross-sectional design, causality could not be established. Additionally, the study was single-centered and based on self-reported data.

**Conclusions:**

The most significant predictor of attitudes toward people with psoriasis was identified as knowledge adequacy.

## Introduction

Psoriasis is a chronic, immune-mediated inflammatory disease characterized by recurring flare-ups over a prolonged period.^1^ Its prevalence varies globally, affecting 125 million people worldwide.^2^, ^3^, ^4^ Psoriasis patients often face a significant psychological burden that can surpass the physical symptoms.^5^, ^6^ It is well-documented that there is an increased incidence of psychiatric disorders such as depression, suicidal ideation, anxiety, sexual dysfunctions, and alcohol dependency in affected individuals.^7^, ^8^, ^9^, ^10^ Moreover, psoriasis patients often receive less social support and may experience greater stigmatization compared to individuals with other dermatological conditions, especially when lesions are located in visible areas such as the face, scalp, and hands.^11^, ^12^, ^13^, ^14^ Social experiences, including exclusion, feelings of worthlessness, rejection, and difficulties in daily life, further exacerbate the impact of psoriasis on patients.

Given the high burden of psoriasis and its negative impact on quality of life, the World Health Organization (WHO) has classified it as a noncommunicable disease of particular importance. In line with WHO recommendations, psoriasis should be regarded with the same level of priority as cancer, cardiovascular diseases, diabetes mellitus, and chronic lung diseases in terms of health services. WHO also advocates for efforts to reduce the stigmatization associated with psoriasis and to raise awareness about the disease.^15^ Additionally, ensuring better access to guideline-based health services for individuals diagnosed with psoriasis is one of the requirements of the United Nations' 2015–2030 Sustainable Development Goals.^16^

Misconceptions about psoriasis can negatively influence attitudes towards individuals diagnosed with psoriasis.^17^, ^18^ Studies conducted in different countries have revealed that a lack of knowledge about psoriasis leads to negative attitudes towards affected individuals.^17^, ^19^, ^20^, ^21^ Health literacy in Turkiye, similar to other underdeveloped countries, is known to be low, and this may further exacerbate negative attitudes toward psoriasis patients.^22^ Moreover, identifying the factors that shape attitudes toward psoriasis is crucial, yet there are limited studies on this topic in these countries. This study aims to evaluate the attitudes, knowledge levels, and associated factors regarding psoriasis among adults in a community-based sample.

## Methods

### Study design and sample

This cross-sectional study was conducted on people aged 18 and over who applied to a university hospital (Eskişehir Osmangazi University Health, Practice and Research Hospital, Eskişehir, Turkey). The local ethics committee approved the study protocol (decision no: 2024/168). This study complies with the Declaration of Helsinki. The sample size of the study was calculated as a minimum of 683 people using the Epi Info program, taking the frequency of having knowledge about psoriasis as 20%, the margin of error as 3% and the confidence interval as 95%. All patients who applied to the hospital as outpatients during working days were included in the study group. 761 people who agreed to participate in the study were reached. To avoid any discriminatory behavior during the study, a questionnaire was administered to 761 people, including 46 patients diagnosed with psoriasis. However, during data analysis, psoriasis patients were excluded, leaving a total of 715 individuals in the final study group.

### Data collection

The questionnaire consisted of three sections. The first part included 17 closed-ended questions, covering the participants' sociodemographic characteristics (10 questions) and independent variables related to psoriasis (7 questions). The presence of physician-diagnosed chronic diseases was inquired about. Participants with diseases other than psoriasis were considered to have a chronic disease. The second part comprised 14 items with three response options (“Yes”, “No”, “I don't know”) to assess participants' knowledge about psoriasis. The third part contained a tool designed to evaluate attitudes toward psoriasis. A 5-point Likert-type scale with 12 items was used to measure these attitudes. The validity of the tool was tested prior to data analysis, and a pilot study was conducted to ensure the clarity and acceptability of all items. Participants completed the questionnaire in approximately 10‒15 minutes.

### Data description

A higher total score on the knowledge questions indicated a greater level of knowledge about psoriasis. The Cronbach’s alpha coefficient for the knowledge questions was calculated at 0.585. Participants who scored above 80% of the maximum possible score (33.6 points or higher out of a total score of 42) were considered to have an adequate knowledge level regarding psoriasis.^23^ For the instrument measuring attitudes toward psoriasis, five items were reverse-coded. As the total score increased, it indicated a more negative attitude toward psoriasis. The possible total score on the scale ranged from 12 to 60.

### Statistical analysis

Statistical analysis performed using SPSS Statistics v15 (SPSS Inc., Chicago, IL, USA) software program. The Cronbach's alpha coefficient, item-total correlation coefficient, and Exploratory Factor Analysis (EFA) were calculated to evaluate the validity of the attitude measurement tool (12 items) towards psoriasis. Cronbach's alpha coefficient of the items was found to be 0.875. Item-total correlation values were between 0.439 and 0.652. When the correlation matrix between the items was examined, it was seen that the correlation coefficient between any two items was not greater than 0.800, so it was accepted that there was no multicollinearity.

The Kaiser-Mayer-Olkin (KMO) test was calculated to evaluate the adequacy of the sample size for Factor Analysis: 0.867, Barlett test: 3607.5751; p < 0.001 was found, so the number of data collected was accepted as sufficient. The item analysis in the EFA of the measurement tool was performed using principal components analysis. The factor loading limit value was accepted as 0.40 (min‒max: 0.609‒0.873). It was found that the model explained 42.4% of the cumulative variance in one dimension. The significant difference between the item scores of the lower and upper 27% groups to evaluate the item discrimination of the scale showed that the discrimination feature of the measurement tool was sufficient (p < 0.001). The measurement tool was accepted as sufficiently valid as a result of the analyses, and the data were analyzed.

The conformity of measurable data to normal distribution was assessed using basic distribution criteria, graphs, and the Kolmogorov-Smirnov test. Since the measurement scores did not show normal distribution, Mann Whitney-*U* and Kruskal-Wallis tests were used in univariate analyses. Spearman correlation analysis was performed to determine the correlation between the scores of the measurement tools used in the study. Multivariate linear regression analysis was applied to determine the predictors associated with the psoriasis attitude scale score. The logarithm of the score was taken to make the psoriasis attitude scale score, which was previously the dependent variable, suitable for normal distribution. A multivariate model was created with variables showing a significance level of p < 0.10 in univariate analysis; p ≤ 0.05 was accepted as the statistical significance value.

## Results

### Characteristics of the study group

Among the 715 participants in the study group, 398 (55.7%) were female and 317 (44.3%) were male. The mean age of the participants was 37.3 ± 13.5 years (range = 18‒78). Seventy-seven percent of the participants had spent most of their lives in urban areas. Sixty-one percent of the participants were university graduates or had a higher level of education, and 53.8% of them were married.

### Psoriasis knowledge questionnaire

Five hundred sixty-three (78.7%) of the participants stated that they had heard of psoriasis before, while 124 (17.3%) reported familiarity with the medical term “psoriasis”. The mean score on the knowledge questionnaire was 32.4 ± 3.7 (median = 33), with scores ranging from 20 to 41.

**Figure 1:**
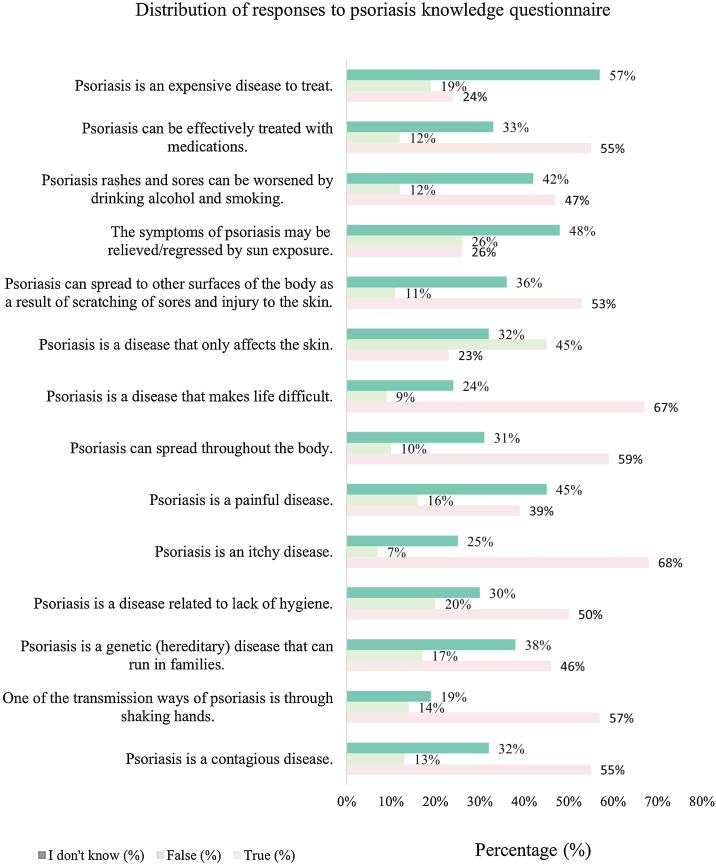
Distribution of responses to the psoriasis knowledge questionnaire in the study group.

Only 284 participants (39.7%) in the study group had an adequate level of knowledge about psoriasis. According to the questionnaire, the most frequently correctly answered items were: “Psoriasis is an itchy disease” (68.0%) and “Psoriasis is a disease that makes life difficult” (67.3%). The least correctly answered items in the questionnaire were “Psoriasis is a disease that only affects the skin” (22.9%) and “The symptoms of psoriasis may be decreased/regressed with sun exposure” (26.0%). The distribution of responses to the psoriasis knowledge questionnaire in the study group is given in Fig. 1.

### Psoriasis attitude scale

The mean score of the Psoriasis Attitude Scale was 30.2 ± 8.4 (median = 30), with scores ranging from 12 to 56. The items with the highest positive attitudes towards psoriasis were “I can be a friend with someone who has psoriasis” (75.7%) and “I would be uncomfortable working in the same workplace with someone who has psoriasis” (69.1%). The items with the lowest agreement were “I can have a sexual relationship with someone who has psoriasis” (36.2%) and “I would swim in the same swimming pool with someone who has psoriasis even if I knew they had rashes” (32.9%). The distribution of responses to the Psoriasis Attitude Scale of the participants is given in Table 1.

**Table 1:** The distribution of the participants' answers to the items related to the psoriasis attitude scale.

Attitude Items	Answers n (%)		
	Agree	Indecisive	Disagree
I would be uncomfortable sitting next to someone who has psoriasis.^a^	96 (13.4)	139 (19.4)	480 (67.2)
I shake hands with someone who has psoriasis.	390 (54.5)	190 (26.6)	135 (18.9)
I would eat with someone who has psoriasis in their home or in a different place.	404 (56.5)	171 (23.9)	140 (19.6)
If I had to marry someone with psoriasis, that would be fine with me.	258 (36.1)	263 (36.8)	194 (27.1)
I can have a sexual relationship with someone who has psoriasis.	219 (30.7)	237 (33.1)	**259 (36.2)**
I can be friends with someone who has psoriasis.	**541 (75.7)**	104 (14.5)	70 (9.8)
I would swim in the same swimming pool with someone who has psoriasis even if I knew they had rashes.	213 (29.8)	267 (37.3)	**235 (32.9)**
As an employer, encountering someone with psoriasis in the workplace would not be a negative factor in my hiring him/her.	456 (63.8)	167 (23.4)	92 (12.8)
I would not want someone with psoriasis to treat me if they have rashes or sores, even if they are a healthcare professional.^a^	145 (20.2)	187 (26.2)	383 (53.6)
I would be uncomfortable working in the same workplace with someone who has psoriasis.^a^	76 (10.6)	145 (20.3)	**494 (69.1)**
I would not allow my child to be in the same room with someone who has psoriasis.^a^	145 (20.3)	161 (22.5)	409 (57.2)
I would not allow my child to marry someone who has psoriasis.^a^	150 (21.0)	243 (34.0)	322 (45.0)

a Negative items.

The median scores of the Psoriasis Attitude Scale were higher among participants who lived in districts or villages, had lower levels of education, were not employed in income-generating jobs, had an income below the minimum wage, had not heard of psoriasis in either the local language or medical terminology, did not have family members or close relatives with psoriasis, and lacked adequate knowledge about psoriasis. In other words, these participants exhibited more negative attitudes. The distribution of the median scores on the Psoriasis Attitude Scale according to sociodemographic and psoriasis-related characteristics is given in Table 2.

**Table 2:** Comparison of scores obtained from psoriasis attitude scale statements in the study group according to sociodemographic and psoriasis related characteristics.

Sociodemographic Characteristics	Psoriasis Attitude Scale Items ScoreMedian (Min‒Max)	Statistical Analysis
		p
**Age group**		
18‒24 age	19 (12‒34)	0.207
25‒34 age	19 (12‒34)	
35‒44 age	18 (12‒32)	
45 age and older	21 (12‒35)	
**Gender**		
Male	20 (12‒35)	0.968
Female	19 (12‒34)	
**Residence**		
City center a	19 (12‒35)	**0.023**
District a	21 (12‒34)	
Village	21 (12‒33)	
**Education**		
University a	18 (12‒34)	**<0.001**
High school	21 (12‒34)	
Primary school	20 (12‒34)	
**Marital status**		
Married	20 (12‒35)	0.532
Not married	19 (12‒34)	
**Income-generating employment status**		
Currently working	18 (12‒35)	**<0.001**
Not working a	21 (12‒34)	
**Family income status**		
Minimum wage and below a	22 (12‒35)	**<0.001**
More than minimum wage	19 (12‒34)	
**Presence of chronic disease**^b^		
Present	20 (12‒34)	0.908
Absent	19 (12‒35)	
**Hearing about “*sedef*”**^c^		
Present	18 (12‒35)	**<0.001**
Absent a	24 (12‒32)	
**Hearing about psoriasis in medical terminology**		
Present	17 (12‒34)	**<0.001**
Absent a	21 (12‒35)	
**Having psoriasis in the family/close relatives**		
Present	17 (12‒34)	**<0.001**
Absent a	21 (12‒35)	
**Knowledge level about psoriasis**		
Adequate	17 (12‒33)	**<0.001**
Not adequate a	21 (12‒35)	
**Total**	19 (12‒35)	

a The category that makes the difference.

b Chronic diseases other than psoriasis.

c This word corresponds to psoriasis in the Turkish language.

**Figure 2:**
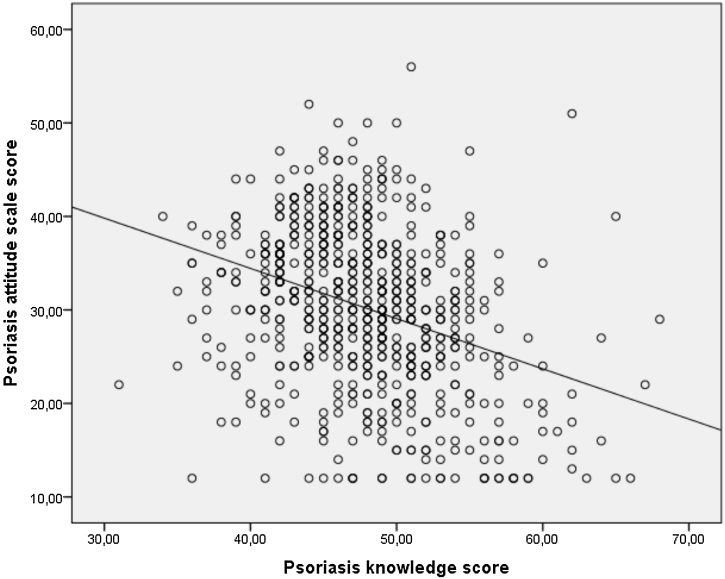
Scatter diagram showing the correlation between the psoriasis attitude scale score and the psoriasis knowledge score in the study group.

A moderate negative correlation was found between the Psoriasis Knowledge Questionnaire scores and the Psoriasis Attitude Scale scores. The scatter diagram showing the correlation between the psoriasis attitude scale score and the psoriasis knowledge questionnaire in the study group is given in Fig. 2.

According to the multivariate linear regression model, factors that predicted a more positive attitude toward psoriasis included working in an income-generating job, having heard of psoriasis in both local and medical terminology, having a family member or close relative diagnosed with psoriasis, and having adequate knowledge about psoriasis. The multivariate model displaying the variables that may affect participants' attitudes toward psoriasis is provided in Table 3.

**Table 3:** Multivariate model showing variables affecting participants' attitudes towards psoriasis.

Variables	The Score of Psoriasis Attitude Scale Items			
	Unstandardized β	Standardized β	(95%CI)	p
Age group	−0.407	−0.304	−1.005‒0.190	0.181
Living in rural area	0.301	0.519	−0.717‒1.319	0.562
Lower educational status	0.953	0.086	−0.017‒1.923	0.054
Working in a job that generates income	−1.812	−0.106	−3.052 ‒ -0.572	**0.004**
Having heard of psoriasis in medical terminology	−3.946	−0.191	−5.374 ‒ -2.518	**<0.001**
Having heard of “*sedef*”	−3.961	−0.178	- 5.518 ‒ -2.404	**<0.001**
Having a family member/close relative diagnosed with psoriasis	−3.961	−0.147	- 4.637 ‒ -1.694	**<0.001**
Having adequate knowledge about psoriasis	−2.880	−0.167	−4.072 ‒ -1.687	**<0.001**
R^2^	0.206			<0.001
F	22.921			

CI, Confidence Interval.

** This word corresponds to psoriasis in the Turkish language.

## Discussion

The present study demonstrated a moderate level of positive societal attitudes towards psoriasis, which were influenced by the participants' knowledge levels. Attitude is defined as a fictional structure representing the degree of liking or disliking of an individual towards a certain attitude object. People's attitude towards a certain attitude object (psoriasis patient) is the positive or negative views that develop towards that object as a result of the integration of thought, emotion and behavioral tendency. It is known that attitudes can be cognitive or affective.^24^ The individual's positive and negative information helps to make decisions about a subject. A positive societal attitude towards psoriasis patients is important as it can reduce the stigmatization of these individuals and lessen the psychological burden of the disease.

It is expected that an adequate level of knowledge about psoriasis in society will reduce misinformation and beliefs about the disease. In this study, the frequency of hearing about psoriasis before in the Turkish language was found to be 78.7% while the frequency of hearing about psoriasis as a term in medical terminology was found to be 17.3%. In the study conducted by Sommer et al., it was reported that 80% of the participants had heard about psoriasis in the German language, while 20% of the participants had heard about the term “psoriasis”.^20^ In the study of Assiri et al., 79% of the study participants had heard about psoriasis in the Arabic language.^25^ It is expected that diseases are known in the local language rather than by their medical terminology. This situation is important in terms of showing that efforts to increase psoriasis literacy in society should be based more on the use of the local language.

In the present study, approximately 60% of participants did not have an adequate level of knowledge about psoriasis. Misconceptions such as psoriasis being a contagious disease (45.8%), caused by a lack of hygiene (49.4%), or transmitted by shaking hands (43.4%) may contribute significantly to the stigmatization of patients. Similarly, a study by Halioua et al. In France reported that 62.4% of the general population lacked knowledge about psoriasis.^26^ In a study by Alzolibani in Saudi Arabia, 45.6% of respondents were unaware of whether psoriasis was contagious or not.^17^ These findings highlight that inadequate knowledge about psoriasis is a widespread issue across different societies.

Positive attitudes towards psoriasis can help reduce the severity of the disease by reducing psychological stress and improving treatment outcomes.^27^, ^28^ First of all, the reliability and validity of the Psoriasis Attitude Scale were tested in this study. Based on these results, the Psoriasis Attitude Scale promised a reliable and valid measurement tool for the Turkish population.^29^ It is noteworthy that 49.4% of the participants scored above the average on the Psoriasis Attitude Scale. Additionally, it was determined that they showed a moderately positive attitude towards psoriasis. In Malaysia, Yong et al. reported at least one misconception towards psoriasis in 64.6% of participants.^30^ Similarly, in a study assessing the perception and stigmatization of people with psoriasis in Germany, it was reported that 59% of the general population had a negative attitude towards people diagnosed with psoriasis.^20^ The varied results in these studies may be explained by using different measurement tools in different cultural societies.^20^, ^30^

In the present study, when examining predictors of attitudes toward psoriasis, educational level and employment in an income-generating job were considered indicators of social status. The authors found that the attitude towards psoriasis was more negative in those participants with lower educational levels. Low educational status may indirectly lead to an inadequate knowledge level and awareness about psoriasis. However, in a study by Almutairi et al., no significant difference was found between educational status and attitudes toward psoriasis.^31^ Other studies have also reported that people's attitudes towards psoriasis are not affected by higher educational levels.^17^, ^32^ Additionally, in this study, a more positive attitude towards psoriasis was found in those who worked in a job that generated income. In contrast, Assiri et al. reported that no significant difference was found between working in a job that generated income and attitudes towards psoriasis.^25^ There are also other studies in the literature that reported no difference between working in a job that generated income and attitudes towards psoriasis.^31^, ^33^ In fact, it can be expected that individuals' attitudes towards differences will be more positive as their interaction with their environment increases in their working lives. The different results presented in the studies may be due to the differences in the educational systems and working environments in the countries and the differences in the measurement tools used.

The present study revealed that those who have a family member diagnosed with psoriasis showed a more positive attitude towards psoriasis. It is predicted that those who are exposed to such health conditions could empathize and also have a more positive attitude towards the situation. Numerous studies have shown that individuals with acquaintances who have skin diseases tend to have a more positive outlook and a higher level of knowledge about the condition.^20^, ^33^, ^34^, ^35^ It has also been reported that those who have a history of personal contact with a psoriatic patient have a more positive attitude towards the disease.^17^

### Study limitations

The present study has several limitations. Due to its cross-sectional nature, a cause-and-effect relationship cannot be demonstrated. Besides, this study was conducted in a single center and relied on self-reported data from participants.

## Conclusion

Approximately 40% of these participants had an adequate knowledge level about psoriasis, and about half showed a positive attitude according to the Psoriasis Attitude Scale. To assess the attitudes toward psoriasis, the authors tested the reliability and validity of the Psoriasis Attitude Scale. It can be used in future studies and may help establish standardization in measuring attitudes across different research. The present study revealed that the most significant predictor of attitudes toward psoriasis was the adequacy of knowledge. This finding suggests that educational interventions and efforts to raise public awareness about psoriasis, particularly by addressing common misconceptions, could play a crucial role in reducing stigmatization.

## ORCID ID

Esra Ağaoğlu: 0000-0001-8985-6224

Bengisu Karagöz: 0009-0007-2092-2044

Hilal Kaya Erdoğan: 0000-0002-8172-1920

Muhammed Fatih Önsüz: 0000-0001-7234-3385

Selma Metintaş: 0000-0002-5002-5041

## Financial support

None declared.

## Authors' contributions

Erman Kavlu: Conceptualization; data curation; formal analysis; investigation; software; visualization; writing-original draft.

Esra Ağaoğlu: Conceptualization; investigation; methodology; writing-original draft; review & editing.

Bengisu Karagöz: Conceptualization; data curation; formal analysis; investigation; software; visualization; writing-original draft.

Hilal Kaya Erdoğan: Conceptualization; investigation; methodology; validation.

M. Fatih Önsüz: Conceptualization; investigation; methodology; resources.

Selma Metintaş: Conceptualization; formal analysis; methodology; project administration; validation; writing-original draft; review & editing.

## Research data availability

The entire dataset supporting the results of this study was published in this article.

## Conflicts of interest

None.
